# AI-driven predictive modeling of sarcopenia progression in limbic-predominant age-related TDP-43 encephalopathy: a novel biomarker fusion approach

**DOI:** 10.1097/MS9.0000000000005037

**Published:** 2026-04-23

**Authors:** Muhammad Mujtaba Chaudhary, Umme Ambreen, Muhammad Tausif, Zain Ul Abedeen, Abad Ur Rehman

**Affiliations:** a Department of Internal Medicine, CMH Institute of Medical Sciences, Bahawalpur, Pakistan; b Department of Internal Medicine, Ayub Medical College, Abbottabad, Pakistan; c Department of Internal Medicine, Saidu Medical College, Swat, Pakistan; d Department of general medicine, Spinghar University (Kabul Campus), Kabul, Afghanistan

*To The Editor*,

Limbic-predominant age-related TDP-43 encephalopathy – neuropathological change (LATE-NC) is a hallmark of LATE, defined by a stereotypical TDP-43 proteinopathy in older adults, with or without coexisting hippocampal sclerosis pathology. LATE is a common TDP-43 proteinopathy associated with an amnestic dementia syndrome, a distinct, slowly-progressive pathology in adults over 80. On autopsy, LATE is often confused with Alzheimer’s disease (AD), which is caused by amyloid plaque buildup; however, the occurrence of co-pathology is not uncommon^[^1^].^ In skeletal muscle biopsies of patients with inclusion body myositis, TDP-43-positive inclusions are found, where sarcoplasmic TDP-43 aggregates link up with mitochondria that are dysfunctional and cause muscle fiber degeneration^[^2^]^. In conjunction with the robust epidemiological association between sarcopenia, diminished gait velocity, and cognitive deterioration, alongside evidence indicating that TDP-43 aggregation impairs mitochondrial function, these findings endorse a biologically plausible *brain–muscle axis* through which LATE-associated TDP-43 pathology may expedite sarcopenia in the elderly population^[^3^]^. Substantial pooled research indicates that both sarcopenia and “possible sarcopenia” approximately double the likelihood of cognitive impairment; nevertheless, dementia was classified as a singular, wide category without neuropathologic stratification, rendering LATE undetectable within the aggregated outcome^[^4^]^.

The mechanistic studies reveal that TDP-43 aggregation alters mitochondrial structure, inhibits oxidative phosphorylation, and activates intrinsic apoptotic pathways, actions contributing to both brain degeneration and skeletal muscle catabolism^[^5^]^. There is strong evidence that resistance training and sufficient protein supplementation are useful strategies for managing and preventing sarcopenia. Additionally, early detection makes it possible to start these focused interventions right away, improving physical performance and lowering the chance of negative consequences^[^6^].^ Traditional syntheses often view sarcopenia as a downstream result of aging, comorbidities, and low-grade inflammation, rather than a potential expression of a particular proteopathy functioning along a central–peripheral axis^[^7^]^.

Major cohorts report the prevalence of LATE-NC as 37% (502/1352 autopsies), with a mean age of 83 years and stages distributed as: 29% stage 1, 58% stage 2, and 13% stage 3. No population incidence rate exists due to autopsy-only diagnosis. Patient population is 45% men (614) and 55% women (738), without gender-stratified prevalence. LATE-NC stages were originally related to cognition and dementia outcomes using robust multilevel logistic and ordinal regression models on NACC data (i.e., generalized linear mixed-effects models and cumulative link mixed models for clustered and ordered outcomes), creating a baseline that was well suited for later machine-learning (ML) fusion but ultimately never implemented for that purpose^[^8^]^.

Furthermore, these evaluations show that traditional regression models are not good at capturing the complex interactions that are likely to occur when neuropathology, genetics, inflammation, and detailed motor phenotypes are all considered together. This suggests that artificial intelligence (AI)-integrated models for such complexities are necessary^[^9^]^.

A biologically plausible muscle-brain axis has been supported by recent observational and Mendelian randomization studies that link sarcopenia and its components (lean mass, muscle strength, and gait speed) to cognitive impairment, with physical activity regulating this association to some extent^[^10^]^. LATE-NC cannot be clinically diagnosed. In 90% of autopsy-confirmed cases, antemortem diagnosis of AD has been received, differentiating them across dementia categories where sarcopenia associations appear significant but unstratified^[^11^]^.

Although LATE is highly prevalent in advanced age and a major contributor to late-life dementia, it has seldom been studied alongside objective measures of muscle mass, grip strength, or gait, leaving its relationship with sarcopenia unclear. This gap raises the possibility of a distinct, TDP-43-driven sarcopenic phenotype rather than nonspecific age-related muscle loss. Addressing it requires integrating LATE-sensitive neuroimaging and emerging TDP-43 biomarkers with genomic, inflammatory, and quantitative body composition and gait metrics within AI-enabled multi-omics models. While definitive TDP-43 biomarkers remain limited and diagnosis is largely postmortem, elevated microtubule-binding region fragment (MTBR-43) in LATE compared with AD offers a promising lead. Combining MTBR-43 with objective muscle and gait measurements may help define a biologically specific sarcopenic phenotype in the oldest-old^[^12,13^]^.

In conclusion, LATE-NC, a prevalent TDP-43 proteinopathy, drives amnestic dementia mimicking AD but cannot be clinically diagnosed antemortem. Consequently, its role in sarcopenia via a plausible brain-muscle axis, i.e., disrupting mitochondrial function in both tissues, has long been overlooked. Sarcopenia doubles the cognitive impairment risk across dementia categories, yet traditional regression models fail to capture these complex neuropathology-muscle interactions. This underscores the need for AI and ML fusion approaches combined with emerging MTBR-43 biomarkers, gait/muscle metrics, and multi-omics to identify a distinct TDP-43-driven sarcopenic phenotype amenable to early resistance training intervention.

## Data Availability

All the data used in the study are available online and can be accessed through the reference list.
